# A Case Report of Reversible Cerebral Vasoconstriction Syndrome in a Patient With Systemic Scleroderma

**DOI:** 10.7759/cureus.24364

**Published:** 2022-04-21

**Authors:** Jieying Liu, Mengni Guo, Richard D Beegle, Ruoyu Miao, Manoucher Manoucheri

**Affiliations:** 1 Internal Medicine, AdventHealth Orlando, Orlando, USA; 2 Radiology, AdventHealth Orlando, Orlando, USA; 3 Hematology and Oncology, Moffitt Cancer Center, Tampa, USA

**Keywords:** calcium channel blocker, steroid, immunosuppressants, systemic scleroderma, reversible cerebral vasoconstriction syndrome

## Abstract

Reversible cerebral vasoconstriction syndrome (RCVS) is represented by recurrent severe thunderclap headache, with or without neurological symptoms. RCVS can be primary or secondary to several factors. Here, we present a case of RCVS in a patient with systemic scleroderma. A 44-year-old female patient presented to the hospital due to Raynaud’s phenomenon, fingertip pain ulceration, skin tightness, and skin depigmentation. She was diagnosed with systemic scleroderma. After four days of steroids, immunosuppressants (mycophenolate mofetil), and hydroxychloroquine, the patient developed severe thunderclap headaches and left lower extremity weakness. The computed tomography angiography (CTA) showed multifocal segmental vasoconstriction of the cerebral arteries. The patient’s headache and body weakness resolved after starting an oral calcium channel blocker (nimodipine).

## Introduction

Reversible cerebral vasoconstriction syndrome (RCVS) is a rare but increasingly recognized disease [1]. This syndrome typically presents with a recurrent severe thunderclap headache, with or without neurological symptoms [2]. Interventional catheter angiography is the gold standard test and will show reversible multifocal segmental vasoconstriction of the cerebral arteries [3]. Why RCVS occurs remains unclear. There are some case reports in patients with systemic lupus erythematosus (SLE) with RCVS [4,5], but reports of RCVS are very limited in patients with systemic scleroderma. Here, we report a case of RCVS in a patient with systemic scleroderma with typical radiological change and a good response to treatment.

This article was previously presented as a meeting abstract at the 2021 Southern Medical Association (SMA) Annual Scientific Assembly on October 29, 2021.

## Case presentation

**Figure 1:**
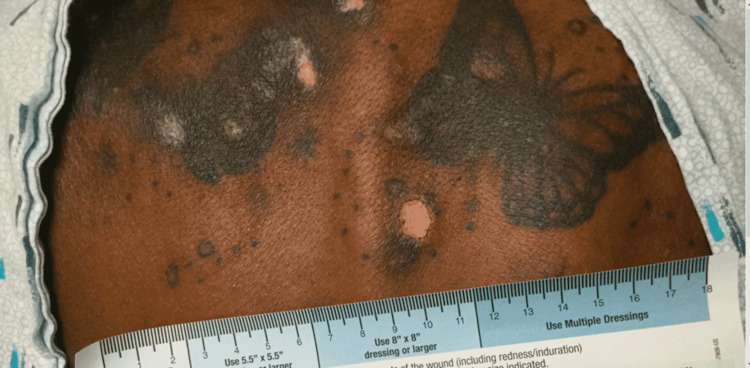
Rash on the patient’s back

A 44-year-old African female with no past medical history presented with Raynaud’s phenomenon, fingertip pain and ulcerations, nail fold capillary changes, facial and back rash, skin tightness and depigmentation (Figure 1), joint pain, morning stiffness, and fatigue. Physical examination showed hypopigmented scarring skin lesions on the face and neck and tenderness and swelling of small joints in the hands and wrists.

The laboratory results were as follows: antinuclear antibody (ANA) + (1:80, speckled), anti-topoisomerase I (anti-Scl-70) antibody + (4.8 U/mL), cardiolipin IgM + (16 MPL U/mL), and smooth muscle antibody (SMA) + (1:20), and anti-Sjögren’s syndrome-related antigen A (anti-Ro/SSA) antibody −, anti-Sjögren’s syndrome type B (anti-La/SSB) antibody −, antinuclear ribonucleoprotein (anti-RNP) antibody −, anti-double-stranded DNA (anti-ds DNA) antibody −, and anti-Smith antibody − (Table 1). Flow cytometry and bone marrow biopsy were negative. The patient was diagnosed with systemic scleroderma. Steroids, mycophenolate mofetil, and hydroxychloroquine were started.

**Table 1:** Laboratory results ANA: antinuclear antibody; anti-Scl-70 antibody: anti-topoisomerase I antibody; anti-Ro/SSA antibody: anti-Sjögren’s syndrome-related antigen A autoantibodies; anti-La/SSB antibody: anti-Sjögren’s syndrome type B antibody; anti-RNP antibody: antinuclear ribonucleoprotein antibody; anti-ds DNA antibody: anti-double-stranded DNA antibody; SMA: smooth muscle antibody

Tests	Results	Reference values
ANA	Positive (titer 1:80, speckled)	Negative
Anti-Scl-70 antibody	4.8 U/mL	<1 U/mL
Anti-Ro/SSA antibody	0.2 U/mL	<1 U/mL
Anti-La/SSB antibody	<0.2 U/mL	<1 U/mL
Anti-RNP antibody	0.5 U/mL	<1 U/mL
Anti-ds DNA antibody	2 IU/mL	<5 IU/mL
Cardiolipin IgM	16 MPL U/mL	<11 MPL U/mL
Anti-Smith antibody	0.2 U/mL	<1 U/mL
SMA	Positive (titer 1:20)	Negative

**Figure 2:**
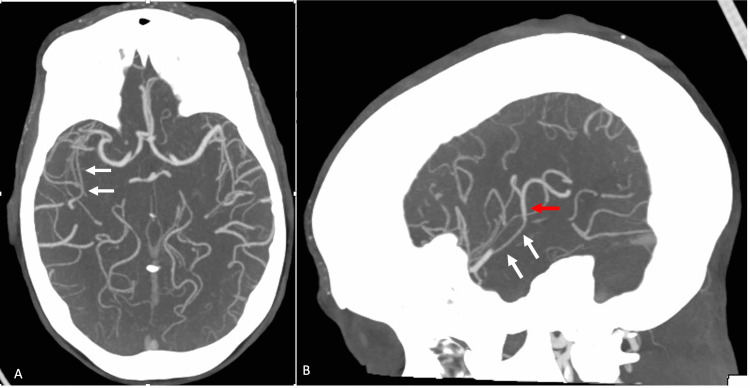
Axial and sagittal maximum intensity projection (MIP) post-contrast CTA images of the head Axial (A) and sagittal (B) maximum intensity projection (MIP) post-contrast CTA images of the head demonstrate a narrowed posterior division M2 branch of the right MCA (white arrows). There is a sharp transition with reconstitution of the normal caliber of the vessel distally (red arrows).

**Figure 3:**
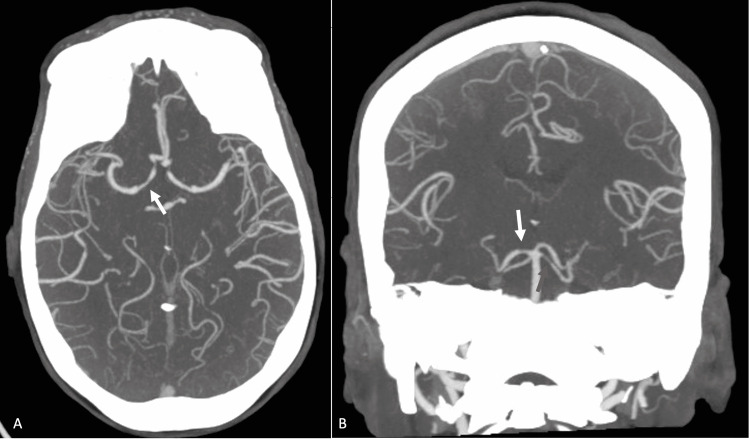
Axial and coronal MIP post-contrast CTA image of the head A: Axial MIP post-contrast CTA image of the head demonstrates a focal narrowing of the A1 segment of the right ACA (white arrow). B: Coronal MIP post-contrast CTA image of the head demonstrates focal narrowing of the P1 segment of the right PCA (white arrow) and focal narrowing of the proximal left superior cerebellar artery (red arrow).

After four days of steroids and immunosuppressants, the patient’s fatigue, joint pain, and fingertip pain improved during the treatment, but she developed a severe thunderclap headache with left lower extremity weakness. A head computed tomography angiography (CTA) was ordered due to concern of vasculitis. The head CTA showed multifocal long segment stenosis ranging from mild to moderate in severity involving the M2 segments of the right MCA (Figure 2A), sharp transition with reconstitution of the normal caliber of the vessel distally (Figure 2B), focal narrowing of the A1 segment of the right ACA (Figure 3A), focal narrowing of the P1 segment of the right PCA (Figure 3B), and focal narrowing of the proximal left superior cerebellar artery (Figure 3B). Neurology was consulted. The clinical presentation and the image findings were consistent with findings of reversible cerebral vasoconstriction syndrome (RCVS).

Based on the RCVS diagnosis as the cause of her headache and lower extremity weakness, nimodipine 60 mg QD PO was started. The patient’s headache resolved; her left lower extremity weakness improved. The patient’s inpatient hospitalization lasted 13 days. After the resolution of the headache and weakness, she was discharged with nimodipine 60 mg QD PO. She has been followed up by her PCP and the rheumatologist for 10 months. She has no more complaints of headaches and extremity weakness.

## Discussion

Approximately 24% of patients with systemic scleroderma with central nervous system involvement present with headaches [6]. Patients with systemic scleroderma have a high incidence of headaches. The estimated prevalence of some of them is believed to be due to migraines [7], but other causes remain unclear. The differential diagnosis and treatment for headaches in patients with systemic scleroderma can be challenging.

RCVS is a unifying term to describe a group of disorders characterized by reversible narrowing and dilatation of the cerebral arteries [3]. There are a lot of risk factors and associated conditions related to RCVS, including vasoactive medications, illicit drugs, and postpartum state [1]. There are also several case reports of RCVS related to SLE and immunosuppressant therapy [3-5,8]. The clinical presentation of RCVS is recurrent sudden, severe thunderclap headaches over several days or weeks. Patients can be with or without focal neurological deficits [3]. The diagnosis of RCVS is based on the recurrent thunderclap headache and typical brain imaging findings, which are reversible multifocal segmental narrowing of the cerebral arteries [9]. The diagnosis of RCVS is difficult to distinguish from cerebral vasculitis for the rheumatology patient, but the symptoms can provide some clues. With cerebral vasculitis, the patient has an insidious onset dull headache and stepwise clinical progression, while the headache of a patient with RCVS is acute, self-limited, and thunderclap in nature. Cerebral vasculitis usually involves the distal cerebral arteries, and the RCVS involves the circle of Willis arteries or their proximal branches [8,10]. Some reports show that calcium channel inhibitors may be a treatment for RCVS [11].

During the systemic scleroderma treatment, our patient developed a severe thunderclap headache and left lower extremity weakness. Our patient may have developed RCVS due to the use of steroids and immunosuppressants or systemic scleroderma. There are reports of RCVS secondary to steroid and immunosuppressant use, such as cyclophosphamide, tacrolimus (FK-506), cyclophosphamide, and interferon-a [8,12,13]. Our patient developed a headache after the use of steroids, mycophenolate mofetil, and hydroxychloroquine; the primary cause of RCVS that we considered should be the use of steroids and immunosuppressants, especially the large dose of steroids [14]. Another pathophysiology we should consider is RCVS due to systemic scleroderma. Lee et al. found that brain-blood barrier (BBB) breakdown was present in 69% of patients with definite RCVS [15]. Patients with RCVS also had impaired cerebral endothelial function [16]. Vascular endothelial injury is also reported in patients with systemic scleroderma. This mechanism may be due to the activation of cellular and humoral immunity [17]. These common mechanisms may explain the development of RCVS in patients with systemic scleroderma.

The antimigraine medication, the steroid, and the immunosuppressant all can cause or worsen RCVS [13,18]. It is important to differentiate RCVS from migraine and vasculitis in patients with systemic scleroderma for the right choice of treatment plan.

## Conclusions

For the patient with systemic scleroderma who developed thunderclap headache, it is necessary to consider RCVS as a differential diagnosis from systemic scleroderma-related migraine and vasculitis. Steroids and immunosuppressants can cause or worsen RCVS. The clinical presentation of RCVS is recurrent sudden, severe thunderclap headaches. The patient can be with or without focal neurological deficits. The diagnosis of RCVS is based on the recurrent thunderclap headache and typical brain imaging findings, which is reversible multifocal segmental narrowing of the cerebral arteries.
